# Supplementary material for: Developing and validating a frailty score based on patient-reported outcome 3 months after stroke: A Riksstroke-based study
Source: PLoS One. 2026 Feb 20;21(2):e0343249. doi: 10.1371/journal.pone.0343249 (PMC12923031; doi:10.1371/journal.pone.0343249)
Supplement: Attachment — Submitted filename: Response to Reviewers.pdf [file pone.0343249.s001.pdf]

Responses to reviewer comment

Reviewer #1: This is an interesting study on a novel concept of using patient reported status to develop a frailty measure.

page 2/15 Introduction - Lines 32 to 42 - This paragraph repeats a significant amount of information in the paragraph immediately above. It appears to be an oversight on the authors part and needs to be consolidated.

Thank you for highlighting this. It was indeed a simple oversight and it is now corrected.

Line 44 - Please list the original reference where the Riksstroke questionnnaire was derived/validated and an internet link to the questionnaire.

Thank you for this important observation. We have added two references for the Riksstroke methodology and a link to the questionnaire.

Why did the authors choose only 9 out of 12 questions from the original patient Rikkstroke questionnaire? why were the questions on a) transportation, b) thoughts, concentration and memory and their effects on ability to carry out daily activities omitted? Impaired cognition is one of several major geriatric syndromes that contribute to frailty. (Edmonton Frail Scale and Frailty Index includes cognition). The authors should justify the omissions especially on the part on cognition.

Regarding item selection, we restricted our analysis to the nine items that map most directly onto the physical and general health domains typical of "frailty phenotypes" (e.g., the Fried criteria). We excluded the question on transportation because it is often influenced by environmental factors (e.g., access to public transport or a car) rather than health status alone. We excluded the question on cognition (thoughts/memory) to focus the score on a coherent physical/physiological frailty construct. While we agree that cognition is a key component of cumulative deficit models (like the Frailty Index), cognitive items often function as a distinct dimension from physical frailty in psychometric analyses. Including them might have violated the dimensionality assumptions or the specific bifactor structure we aimed to establish. We have added a justification for this selection by the end of the first paragraph in the Material and methods - Riksstroke subsection and listed the exclusion of cognition as a limitation in the Discussion.

page 6/15 - Can the authors describe the basic or simple aspects of the results further. For example , what is the range of possible scores. (minimum to maximum) of this PROM scale? What is the score range of the first and second quartile (which seem to have similar mortality rates), and score range of the third and fourth quartile? Can any of these quartiles be equated to no frailty, mild, moderate and severe frailty? If this cannot be done for the time being and needs further validation studies against standardised tools, then the authors can explain as such.

We appreciate this suggestion and agree that this could have been more clear. We have updated the Results section (last paragraph under score fairness) to state the observed range of the frailty score (θ).

Regarding the quartiles: We have added the specific score boundaries for Q1–Q4 in the text by the end of the first paragraph under Results-external validity. However, we have refrained from explicitly labeling them as "mild," "moderate," or "severe" at this stage. As now noted in our Discussion, establishing clinical cut-offs requires calibration against external clinical standards or established tools, which was outside the scope of this registry-based study. We have clarified this distinction in the revised manuscript.

In the discussion section, can the authors add in discussion about the potential future clinical utility of this frailty measurement tool? Eg a risk stratification table to estimate 3 year survival rate perhaps? And future studies to correlate with care needs and health care resources utility. Many readers will be clinicians who have some basic statistical knowledge but the main points of interest would be the daily use of this frailty assessment tool in the care plan of the person living with stroke.

We appreciate this suggestion to bridge the gap between statistical validation and daily clinical practice. We agree that the ultimate value of this score lies in its application to care planning and risk stratification. We have added a new paragraph before the last paragraph in the Discussion section highlighting how the clear separation in survival curves (Figure 3) supports the future development of probability tables for 3-year survival to aid prognostication. We have added text suggesting that high PROM-based frailty scores could act as a "red flag" to trigger Comprehensive Geriatric Assessment or review of home-care needs. Additionally, we have added a recommendation for future studies to link this score to healthcare utilization data (readmissions, institutionalization) to validate its use in resource allocation.

page 8/15 - Table 5 - Are the terms two-factor and bifactor standardised statistical terms? Although they are explained further on in the manuscript, it can be confusing to the reader as they are close in meaning in English. If possible the authors can consider changing one of the terms eg two-factor.

We agree that the similarity in terms can be confusing to readers less familiar with psychometrics. While "Two-factor" (confirmatory factor analysis) and "Bifactor" are standard technical terms, we have renamed the "Two-factor model" to **"***Correlated-factors model***"** *throughout the text and tables to clearly distinguish it from the Bifactor model and avoid ambiguity.*

page 11/15 - Line 286 - .....1 100-day follow up....., do the authors mean the first 100 days follow up?

We meant the 1100 days for which we tracked the individuals after they completed the questionnaire, but this was not clear. We have added some clarification on this under both the "Material and methods - external validity" and the "Results - external validity" sections.

Reviewer #2: I think the authors have thoroughly assessed and validated a frailty score based on patient reporte data. Based on my limited experience in this field of prediction score creation, this study sounds safe to be accepted.

Thank you for looking at our manuscript.
